# Functionalized sodium alginate composite films based on double-encapsulated essential oil of wampee nanoparticles: a green preservation material

**DOI:** 10.1016/j.fochx.2024.101842

**Published:** 2024-09-19

**Authors:** Jinman He, Wanli Zhang, Gulden Goksen, Mohammad Rizwan Khan, Naushad Ahmad, Xinli Cong

**Affiliations:** a School of Life and Health Sciences, Hainan Province Key Laboratory of One Health, Collaborative Innovation Center of One Health, Hainan University, Haikou 570228, PR China; b School of Food Science and Engineering, Hainan University, Haikou 570228, PR China; c Department of Food Technology, Vocational School of Technical Sciences at Mersin Tarsus Organized Industrial Zone, Tarsus University, Turkey; d Department of Chemistry, College of Science, King Saud University, Riyadh 11451, Saudi Arabia.

**Keywords:** Essential oil, Nanoparticles, Film properties, Preservation application, Pectin (PubChem CID:854), Sodium alginate (PubChem CID:5102882), Ethanol (PubChem CID:702), Glycerol (PubChem CID:753).

## Abstract

In this study, zein-pectin nanoparticles loaded with Wampee [*Clausena lansium (Lour.)* Skeels] (WEO) were developed. The particle size of the nanoparticles is 515.9 nm, polydispersity index is 0.4 and zeta potential is −39.3 mV. Subsequently, the ZWP was incorporated into sodium alginate (SA)-based film (ZWP-S). The films were then analyzed to determine their physical properties and thermal stability, and also to examine their microstructure and intermolecular forces using SEM, FTIR, and XRD techniques. Additionally, the films were evaluated for their antimicrobial and antioxidant activity, as well as their ability to sustain the release of WEO. Overall, the ZWP-S film conferred excellent functional properties, including UV barrier performance, mechanical properties (21 % increase in tensile strength), water sensitivity, stability, more compact structure, high antioxidant activity and long-lasting antimicrobial activity, surpassing those of the control film. Consequently, it was applied as a novel coating for preserving strawberries, rotting rate of strawberries was reduced by 43 % at 6d, yielding promising results in prolonging the freshness of the fruit.

## Highlights

• Sodium alginate-based films incorporating nanoparticles (ZWP-S) were prepared.
• The possible encapsulation mechanism in the ZWP-S films delayed the release of WEO.
• ZWP-S films deliver more sustained bacteriostatic activity.
• The ZWP-S films showed interesting prospects for fruit preservation.

## Graphical abstract

**Figure:**
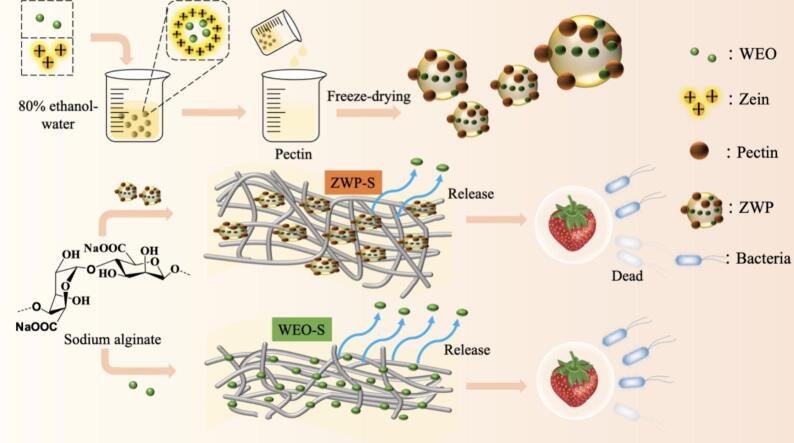

## 1 Introduction

Traditionally, the primary function of food packaging is to maintain the quality and safety of food by protecting it from contamination from physical, chemical and biological sources. Currently, conventional packaging and chemical preservation methods continue to be the main forms of technology in the food packaging industry. The majority of conventional packaging materials are synthetic polymers made from petroleum-based chemicals. However, these synthetic polymers are typically not capable of being broken down naturally (Zhang et al., 2024).

Sodium alginate (SA) is one of the natural materials currently being focused on for biodegradable food packaging (Yan, Lan, & Xie, 2023). SA is a naturally occurring polymer produced from macroalgae or fungi, such as kelp. It is known to be highly safe and non-toxic for both the human body and the environment. It possesses excellent solubility, moisturizing properties, dispersion, film-forming ability, and air permeability. SA regulates the ratio of gas exchange and water evaporation in packed food, which suppresses and slows down respiration and metabolism. Additionally, it possesses specific viscosity and gel qualities that can efficiently resist bacterial invasion and prevent microbial recontamination or loss of food quality (Ebrahimzadeh, Biswas, Roy, & McClements, 2023). However, in comparison to traditional polyethylene plastic films, simple SA-based films often have certain shortcomings in terms of barrier and mechanical characteristics. Therefore, it is typically necessary to choose appropriate modifiers and utilize their interactions with SA-based films to improve the various properties of the films (Zhang, Jiang, Rhim, Cao, & Jiang, 2022).

Essential oil (EO) appear to be promising natural plant-based improvers. For example, the EO of Wampee (*Clausena lansium [Lour.] Skeels*) (WEO) is derived from a plant in the Brassicaceae family, which is a widely available plant material in tropical China and Southeast Asian countries. Researchers have discovered a large number of biologically active components, such as phenolics, flavonoids, coumarins, and amides, in the WEO (Chang et al., 2018). Also, it has excellent antioxidant, antimicrobial, anti-inflammatory, anti-obesity and antitumor activities (Arbab et al., 2012). For example, antifungal activity of the EO of *Clausena lansium* (*Lour.*) *Skeels* seeds had been demonstrated against *Candida albicans* in a previous report (Ma et al., 2023). Nevertheless, similar to the majority of plant essential oils, the characteristics of WEO, such as its susceptibility to degradation by light and heat, as well as its limited ability to dissolve in water, restrict its usage as a modifier in films. This study suggests that encapsulating essential oils (EOs) within a biopolymer matrix is a feasible method for stabilizing the active agent and facilitating controlled release of the active compounds (Zhang, Jiang, et al., 2022). Specifically, it is reported that zein is proposed as a matrix for encapsulation, but individual zein nanoparticles exhibit inadequate stabilization and the lowest loading and encapsulation rates (Kasaai, 2018). Researchers have shown that zein-polysaccharide nanoparticles provide better stability and protection than individual zein nanoparticles (Yuan, Ma, Xu, & Wang, 2022). For example, Elmizadeh, Goli, Mohammadifar, and Rahimmalek (2024) indicated that the increased interaction between pectin and zein, involving hydrophobic, hydrogen bonding, and electrostatic interactions, increased the effective encapsulation of curcumin from 50.62 % to 78.83 % using appropriate concentrations. Zein proteoglycan nanoparticles improved stable delivery of the active substance and also developed its sustainable release. According to another study, konjac glucomannan (KGM) films that were activated by zein and pectin nanoparticles, which are stabilized with oregano EO Pickering emulsion (ZPEO), were able to release oregano EO at a slow rate for a period of 21 days or more (Zhang, Cao, & Jiang, 2022). Currently, to the best of our knowledge, the effect of nanoparticles prepared from zein and pectin co-encapsulated with WEO on the performance of SA films has not been reported. Furthermore, there have been no findings on the incorporation of composite nanoparticles containing encapsulated water-in-oil emulsion (WEO) into functionalized edible coatings based on SA for the purpose of fruit preservation. Therefore, in this study, a novel freshness preservation film material was developed by introducing composite nanoparticles encapsulating WEO using SA as a film-forming matrix. Firstly, nanoparticles of zein and pectin co-encapsulated with WEO were prepared, and secondly, the effects of direct addition of EO and encapsulated EO on the physical, slow and controlled release, antibacterial, and antioxidant properties of the SA-based film were investigated. Finally, the effect of functionalized SA-based composite films on strawberry preservation was explored.

## 2 Materials and methods

### 2.1 Materials

The WEO was extracted from wampee leaves grown at Hainan University campus. After GC–MS analysis, the main components: β-Caryophyllene oxide (12.73 %), humulene epoxide (11.38 %), curcumin (8.54 %), etc. Zein (purity >98 %), pectin, sodium alginate, and glycerol were purchased from Shanghai Aladdin Biochemical Technology Co., Ltd., as analytical grade. Strawberries were picked from a local orchard (Hainan, China) and transported to the laboratory for application experiments. The *Escherichia coli* (*E.coli*) and *Staphylococcus aureus* (*S. aureus*) for antibacterial assay were obtained from China General Microbiological Culture Collection Center. Ultrapure water was used for all experiments.

### 2.2 Preparation of nano-composite particles

This process was carried out in order to produce ZWP nanoparticles following the method by Elmizadeh et al. (2024) with slight modifications. Briefly, zein was dissolved in 80 % ethanol-water solution (4 %, *w*/*v*). Subsequently, the WEO (0.15 %, *v*/v) was gradually added to the solution. Additionally, an aqueous pectin solution (2 %, w/v) was prepared and then utilized in the antisolvent precipitation method. All dispersions were frozen at −20 °C overnight and lyophilized by a freeze drying system at −50 °C for 48 h.

### 2.3 Particle size, polydispersity index (PDI), zeta potential determination and encapsulation effciency (EE)

Particle size, PDI, zeta potential determination: the sample was diluted with ultrapure water (0.01 g L^−1^) and measured by a laser particle size analyzer (zetasizer nano ZS90, Malvern Instrument).

The method of Khan, Chen, and Liang (2021) and Chen et al. (2020). with some modifications, in brief, the nanoparticles were mixed with ethanol and stirred, the WEO absorbance was determined at 278 nm using an enzyme marker (Tecan/Infinite E Plex, Switzerland). The WEO content was calculated from the established standard curve. The linear regression equation was Y=0.0085X+0.0259, R^2^ = 0.9977, where X and Y were the WEO concentration and the corresponding absorbance, respectively. The EE of the nanoparticles was calculated:

(1) $$\mathrm{EE}\%=\frac{\text{Total}\;\mathrm{Res}-\text{Free}\;\mathrm{Res}}{\text{Total}\;\mathrm{Res}}\times 100$$

### 2.4 Preparation of films

The films were fabricated using our previous method (He et al., 2024). Initially, SA was dissolved in an aqueous solution (2 % *w*/w) with magnetic stirring (800 rpm) and completely dissolved after 24 h. For ZWP- SA film-forming solution, 50 % ZWP (*w*/w SA) was uniformly dispersed in 50 mL of aqueous solution (800 rpm, 30 min) before being introduced into 45 mL of SA solution, denoted as ZWP-S group. The WEO—SA film-forming solution (WEO, 0.15 % *v*/v) was prepared by dissolving the WEO in 5 mL of 80 % aqueous ethanol and then directly added to the 95 mL of SA solution was prepared and denoted as WEO-S group. To reduce the error, 5 mL of 80 % aqueous ethanol solution was also added to the other groups. All groups were added with 30 % glycerol (w/w SA) as a plasticiser and then mechanically homogenised (9000 rpm, 2 h). Finally, it was sonicated for 30 min and degassed. The final SA concentration for all groups was maintained at 2 % (*w*/w). 20 mL of homogeneous film-forming solution was slowly poured into a polystyrene plastic petri dish and dried at 60 °C for 10 h to create films. The films were placed in moisture balance for 48 h at 25 ± 0.5 °C and 65 ± 5 % relative humidity.

### 2.5 Characterization of films

#### 2.5.1 Micro-morphology and appearance of films

Scanning electron microscope (SEM) (TESCAN MIRA, Czech Republic) was used to observe the surface and cross-sectional morphology of the films. The application of liquid nitrogen embrittlement was followed by gold spraying, and then these films were scanned and examined at an accelerating voltage of 5 kV.

#### 2.5.2 Thickness, color and light transmission

The thickness of the films was measured and averaged by a spiral micrometer at nine random locations on the composite film. The *L*, *a*, and *b* values of the films were determined after standard white plate correction using a colorimeter, and the total color difference *∆E* was calculated according to eq. (2):

(2) $$△E=\sqrt{{\left(L-L_{0}\right)}^{2}+{\left(a-a_{0}\right)}^{2}+{\left(b-b_{0}\right)}^{2}}$$

where *L*, *a*, *b*- are film color parameters; *L*_*0*_, *a*_0_, *b*_*0*_- are whiteboard measurements.

The transmittance spectra of the films were detected in the wavelength range of 200–800 nm using an UV spectrophotometer (UV1600PC, Shimadzu, Japan). The opacity was calculated as following equation:

(3) $$\text{Opacity}=\frac{A_{600}}{T}$$

#### 2.5.3 FTIR spectroscopy

The chemical structure of the films was analyzed using FTIR (Thermo Nicolet iS5, Thermo Fisher, US) to investigate the interaction between the sodium alginate matrix and both WEO and nanoparticles. The scanning resolution was 4 cm^−1^ within the wavelength range of 4000–400 cm^−1^, and a total of 32 scans were performed.

#### 2.5.4 X-ray diffraction (XRD)

XRD spectra were collected using an X-ray diffractometer (D8 Advance, Bruker, Germany) operating at 40 kV, 20 mA, rate: 4° min^−1^, 10–80° (2θ).

#### 2.5.5 Mechanical properties

The mechanical properties encompass two key parameters: tensile strength (TS) and elongation at break (EB). They were measured using a texture analyzer (TA. XT plus, Stable Micro Systems, UK). The film sample has a rectangular form with dimensions of 1 cm × 5 cm. The initial distance between the film ends is 30 mm, and the film is pulled at a speed of 0.5 mm s^−1^ until it ruptures. These parameters were computed using the following equations:

(4) $$\mathrm{TS}\left(\mathrm{MPa}\right)=\frac{F}{S}$$

(5) $$\mathrm{EB}\left(\%\right)=\frac{L_{1}-L_{0}}{L_{0}}\times 100$$

Among them, F: maximum tension (N); S: cross-sectional area (mm^2^); L_1_: final length (mm); L_0_: initial length (mm).

#### 2.5.6 Thermogravimetry (TGA) analysis

The thermal denaturation temperature for samples was determined by thermogravimetric analyzer (NETZSCH TG 209F3, Germany). A certain amount of the samples was weighed and sealed in an aluminum tray. The temperature rise rate was set to 10 °C min^−1^, within the range of 30–600 °C.

#### 2.5.7 Moisture content (MC), swelling ratio (SR), water contact angle (WCA) and water vapor permeability (WVP)

The film samples were weighed at room temperature as M_1_, and then dried at 105 °C until the mass was unchanged, which was recorded as M_2_, and calculated according to the following eq.

(6) $$\mathrm{MC}\left(\%\right)=\frac{M_{1}-M_{2}}{M_{1}}\times 100$$

The film samples were weighed at room temperature for mass M_3_, immersed in 50 mL of ultrapure water, wiped for residual water on the film surface and then weighed and recorded as M_4_ to calculate the swelling ratio of the film.

(7) $$\mathrm{SR}=\frac{M_{4}-M_{3}}{M_{3}}\;$$

Surface hydrophobicity was measured by a contact angle analyzer (OSA100C, Ningbo, China). Film strips were placed flat on slides, and distilled water (20 μL) was dropped on the sample surface using a micro syringe. The average values at different locations on the film surface were computed.

The WVP was determined according to the previous methodology (Zhang, Cao, & Jiang, 2022). Each film was sealed in a conical flask containing 5 g of anhydrous calcium chloride. The cups were placed in a desiccator at constant temperature and humidity and their weights were monitored every 24 h and stored for 3 days.

(8) $$\mathrm{WVP}=\frac{∆m\times d}{A\times t\times ∆p}$$

where, Δm, d, A, t and Δp correspond to the moisture weight gain of the sample (g), the mean value of the film thickness (mm), the area of the film exposed to moisture transfer (m^2^), the penetration time (s) and the difference in water vapor pressure between the two sides (Pa).

#### 2.5.8 Antioxidant properties

Antioxidant activity properties according to the DPPH and ABTS radical scavenging capacity of all samples was assessed with our previous method (He et al., 2024).

#### 2.5.9 Antimicrobial activity

The antimicrobial activity of the film against *Escherichia coli* (*E. coli*) and *Staphylococcus aureus* (*S. aureus*) was detected (Zhang et al., 2022). The strains were preactivated before use, and the bacterial suspension was obtained by culturing at 37 °C for 12 h. The sterilized film samples (2 cm × 2 cm) were prepared, added to 50 mL of LB medium and 0.5 mL of bacterial suspension, and the OD_600_ value was measured every 3 h until 24 h. Finally, the bacterial suspension was taken and spread on agar plates to record the bacterial growth status. The control group was an equal volume of bacterial suspension, and the blank was pure medium.

#### 2.5.10 Releasability

An aqueous food system was simulated with 10 % ethanol according to the method of Fan et al. (2023) with slight modifications. The films were cut into 2 cm × 2 cm pieces and fully submerged in a sealed, dark bottle containing 50 mL of the simulant. The absorbance of WEO was measured at different time intervals, at 278 nm. The concentration of the released substance was appointed by utilizing a previously constructed WEO-ethanol standard curve. The rate of release (RR) was expressed in the below eq.

(9) $$\mathrm{RR}\;\left(\%\right)=\frac{C}{C_{0}}\times 100.$$

where C_0_, C- are WEO content of the film and release content.

### 2.6 Strawberry coating preservation

Fresh strawberries that were washed and had undamaged surfaces were completely immersed in SA, WEO—S, and ZWP-S solutions (all solution at 23 ± 0.5 °C) for 1 to 2 s, respectively. Then, the strawberries were naturally dried. Each group was randomly assigned 30 strawberries, changes in the appearance of the strawberries were recorded, and the quality indicators (weight loss, rotting rate, and decay index) were measured daily. In addition, in vivo changes of the strawberries was observed on 6d. Some of the indicators were measured as follows:

Weight loss was determined using the weighing method (Khalifa, Barakat, El-Mansy, & Soliman, 2016). Weight loss was expressed in the below equation.

(10) $$\text{Weight loss}\left(\%\right)=\frac{\text{the initial weight}-\text{the current weight}\;}{\text{the initial weight}}\times 100$$

Rotting rate was derived as a proportion of the number of rotted individuals to the total number of strawberries.

The decay index is based on the degree of surface decay and is classified on a scale of 0 to 5 (0: no decay; 1: ≤ 20 %; 2: 20–40 %; 3: 40–60 %; 4: 60–80 %; 5: ≥ 80 %). The decay index was expressed in the below equation.

(11) $$\text{Decay index}\left(\%\right)=\frac{\sum \left(\text{the decay grade}\times \text{number}\right)}{\text{the highest grade}\times \text{total number}}\times 100$$

### 2.7 Statistical analysis

The data was processed and plotted using IBM SPSS Statistics 25 and Origin 19 software, respectively. One-way analysis of variance (ANOVA) was used and the Duncan test was chosen to assess the significance of differences (*p* < 0.05). The results were expressed as mean ± standard deviation (SD).

## 3 Results and discussion

### 3.1 Nanoparticle size, zeta potential, PDI and EE

As shown in Table 1, the ZWP nanoparticles were uniformly distributed with a PDI of 0.4 and narrow polydispersity (PDI < 1), which also indicated excellent redispersibility in aqueous solution. The particle size of ZWP is 515.9 nm. Similarly, Wang et al. (2021) noted that polysaccharides improved the high hydrophobicity of zein nanoparticles and reduced particle aggregation. The zeta potential of the ZWP nanoparticles was −39.3 mV (greater than ±30 mV), which suggested that the nanoparticles could be dispersed stably and homogeneously in the medium by electrostatic repulsive interactions (Mo et al., 2021). The level of EE directly affects the ability of nanoparticles to effectively protect the active ingredients. The EE of ZWP nanoparticles reached 89.7 %, indicating that the desirability of the preparation method in this study. In a recent report, the EE of pectin-zein containing tanshinone prepared using the antisolvent precipitation method increased from 50.52 % to 79.41 % compared to nanoparticles without pectin (Elmizadeh et al., 2024).

### 3.2 Film characterization

#### 3.2.1 Morphology

**Fig. 1:**
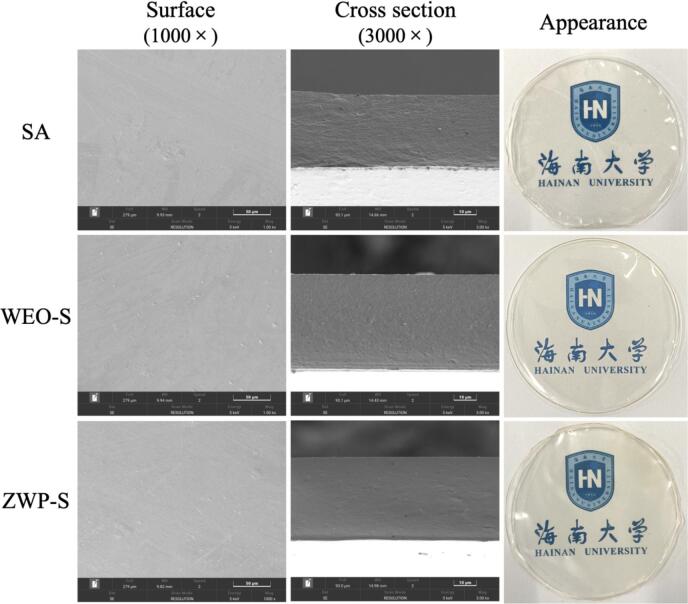
SEM of the surfaces and cross-sections of the films and photos of the appearance.

The microstructure of the films and the distribution of WEO and nanoparticles in the film matrix were analyzed by SEM. Fig. 1 revealed that the films did not exhibit any significant cracks and displayed a uniform and smooth material. This can be ascribed to the exceptional film-forming characteristics of the SA matrix. The presence of some spherical oil droplets and a few small pores, uniformly dispersed in the substrate, were observed in the WEO-S films. However, there was no delamination or large pore structure, indicating that the WEO had been successfully loaded into the film. A similar phenomenon was also found by Liu et al. (2023) in the wampee seed EO-chitosan-based films, where the “particulate matter” in the ZWP-S films was significantly less, which could be attributed to the excellent miscibility between the SA matrix and the ZWP, and the stronger electrostatic interactions between the two resulting in a homogeneous structure and a more compact arrangement. Furthermore, the films encapsulating EO with nanoparticles have the ability to decrease the size of small openings and clusters that are generated as a result of the volatilization, agglomeration, flocculation and emulsification of EO during drying. Additionally, these films also result in a smoother surface. Externally, almost all films possess the characteristics of being flat and transparent.

#### 3.2.2 Thickness, color, and light transmission

Thickness results were indicated in Table 2, the SA film (0.074 ± 0.004 mm) showed the highest value of thickness, while with a slight decrease in the thickness of the WEO-S film (0.066 ± 0.006 mm) and the ZWP-S film (0.068 ± 0.006 mm). It may be due to the loss of the oil phase during the film production process, which may lead to a decrease in the concentration of solids in the film matrix (Acevedo-Fani, Salvia-Trujillo, Rojas-Graü, & Martín-Belloso, 2015). Khachani et al. (2024) detected that the addition of *Tthymus capitatus* and *Cinnamomum verum* EOs to the pectin film-forming matrix reduced the film thickness. In addition, the incorporation of 2.5 and 5 % *Marjoram* EO loaded nanoemulsion (*p* < 0.05) also significantly reduced the film thickness values (Almasi, Azizi, & Amjadi, 2020).

**Fig. 2:**
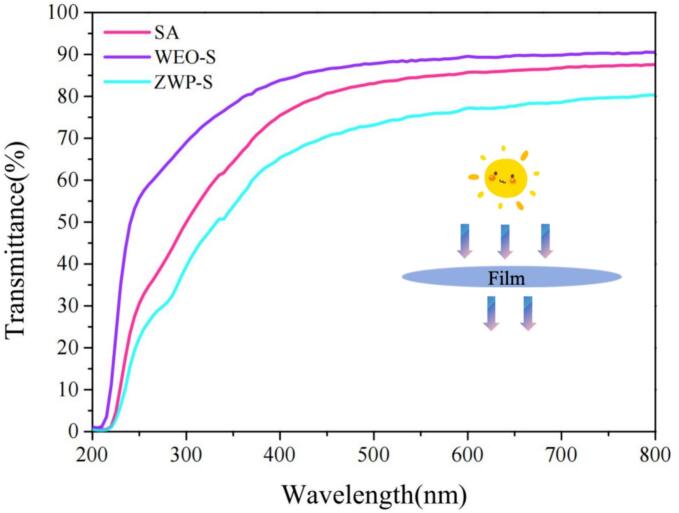
UV–vis light transmittance spectra of the films.

The color of food packaging films affects consumer acceptance and decisions regarding consumption. The impact on the color properties of the films was represented in Table 2, where, in general, all films found a light color (*L*-value >70) with no significant change in the a-value, and, unexpectedly, the WEO-S film showed a slightly larger *L*-value than the SA film alone. To the best of our knowledge, the incorporation of thymol into SA matrix in the study of Wei et al. (2023), similarly reported an increase in brightness. On the other hand, the ZWP-S films exhibited significantly lower *L*-value and ∆*E*, with the greatest *b* value (−0.41 ± 0.65), suggesting a slight increment in color yellowness. This might be explained to the inherent yellowish color of the nanoparticle shell material. . Moreover, the inclusion of WEO did not have significant effect on the transparency of the SA film, whereas the presence of ZWP nanoparticles substantially raised the opacity of film. The UV–Vis transmittance of the films (Fig. 2) also supported this observation, with the ZWP-S films exposing significant UV-protective behavior as a result of presenting low transmittance in the UV and visible regions. In a previous study, cinnamon essential oil-loaded Pickering emulsion functionalized composite films stabilized by zein-pectin nanoparticles with reduced light transmission (Wu et al., 2023).

#### 3.2.3 Chemical structure of the films

**Fig. 3:**
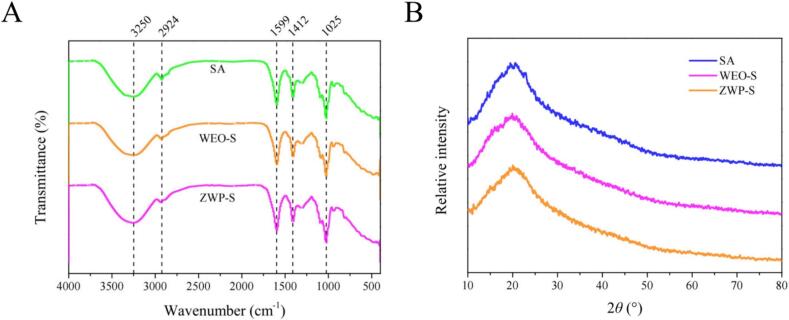
FTIR (A) and XRD (B) analysis of the films.

FT-IR analysis was conducted to determine if any alterations were made to the existing bonds in the SA film as a result of the introduction of new components, or if any new chemical bonds were created between the SA network and the added compounds. The IR spectra were obtained for SA, WEO-S and ZWP-S films. In Fig. 3A, all films displayed the characteristic absorption peaks near 3250 cm^−1^, near 2924 cm^−1^, and near 1700 to 800 cm^−1^. The absorption peaks near 3250 cm^−1^ were generated by O—H symmetric and asymmetric telescopic vibrations; the vibrational peaks of C=O at 1599 cm^−1^, and the characteristic absorption peaks of the polysaccharides from 1700 to 800 cm^−1^, the interaction between the substrate directly with WEO, or the nanoparticles encapsulating the WEO.They were predicted to be non-covalent, as not much change in the FTIR peaks of the films was noted, except for the change in the peak intensity and the peak position. Li et al. (2022) similarly pointed out that physical interactions occurring among the components in the zein/chitosan films containing *Mosla chinensis* ‘Jiangxiangru’ EO resulted in differences in peak intensities without the formation of covalent bonds.

The films were analyzed by XRD and Fig. 3B illustrated that the structure of the SA films was predominantly amorphous, with broad-band characteristic peaks at around 2θ = 15–30°. Upon the inclusion of WEO and ZWP, all samples exhibited distinct peaks with varying intensities at comparable positions, indicating the interactions between WEO, ZWP, and SA. These interactions caused dispersion within the SA matrix, leading to the disruption of its original structure. However, no disparity in the crystallinity of the films was detected. In overall, the XRD patterns confirmed a high level of compatibility among the film-forming polymers, including SA, WEO, and nanoparticles.

#### 3.2.4 Mechanical properties of the films

**Fig. 4:**
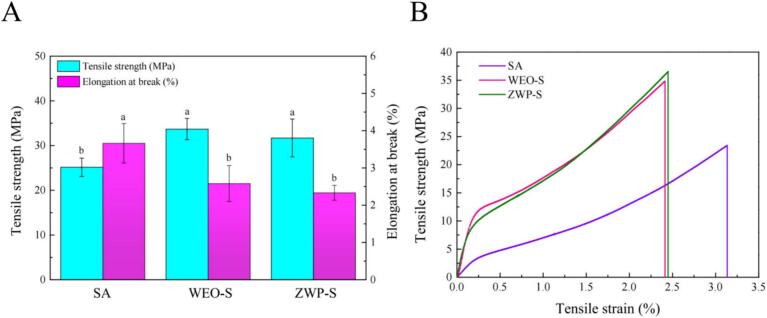
TS, EB (A) and typical stress curves (B) of the films.

There were significant differences in TS and EB between SA films and films with WEO and ZWP added (Fig. 4). In fact, a previous report indicated that the mechanical strength of the film decreased when various EOs such as *R. officinalis* L, *A. herba alba Asso, O. basilicum* L, and *M. pulegium* L were incorporated into the films. (Mahcene et al., 2020). The TS of the film was usually reduced by 45–70 %, depending on the type of oil. In the presence of EO, the mechanical properties of the film were largely reduced (Ebrahimzadeh et al., 2023). In the present study, unexpectedly, the addition of WEO increased the TS of the films while EB decreased, and the improvement in the mechanical properties could be attributed to the formation of intermolecular H-bonds between the polymer and WEO, as well as the good distribution and compatibility between them. The addition of ZWP blended in SA films increased the TS at fracture from 25 MPa to 33 MPa and declined the EB from 3.66 % to 2.33 %, respectively. The reason may be that the nanoparticles were incorporated, resulting in the polymer chains in the film being entangled with each other and the free volume was reduced, with the consequent improvement of TS. As a result, ZWP-S could withstand greater stresses than SA films (Oymaci & Altinkaya, 2016). The mechanical properties of zein films with the addition of cinnamaldehyde and acetyl eugenol were also significantly boosted (Kaur & Santhiya, 2021).

#### 3.2.5 Thermogravimetric analysis

**Fig. 5:**
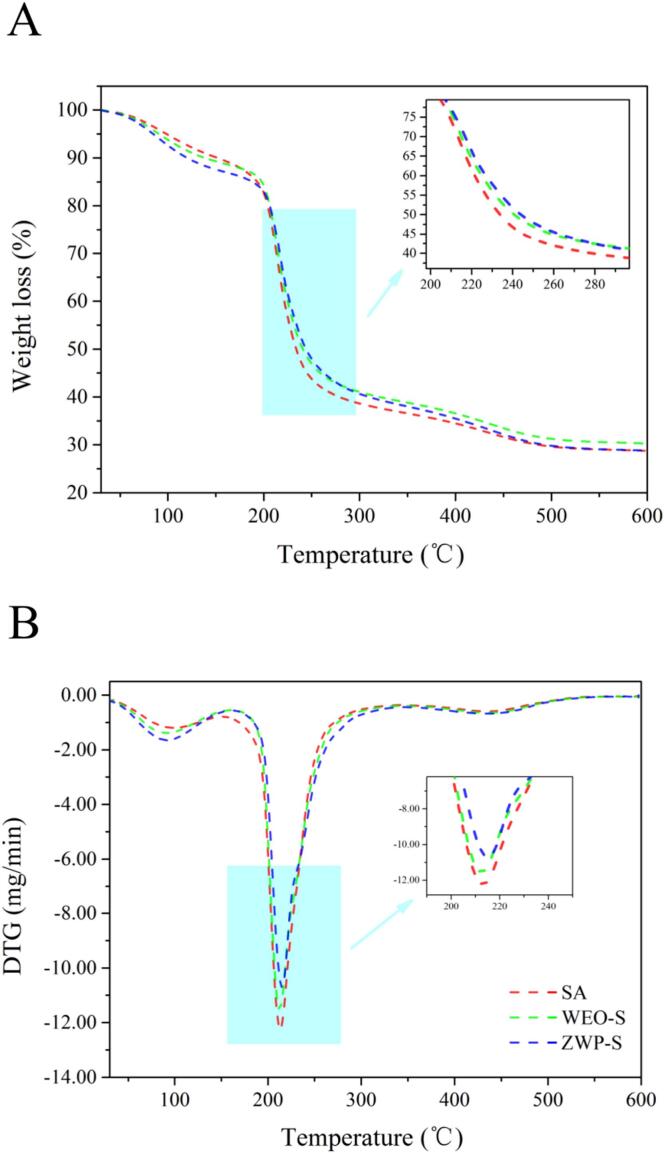
TGA thermograms (A) and DTG (B) of the films.

In Fig. 5A. Thermal degradation of the films occurred in two distinct phases. The first phase was carried out between approximately 100–180 °C for the thermal degradation of the aromatic components of the films evaporated at low boiling with water. A similar weight loss of less than 15 % was observed for all films. The second phase of weight loss took place at temperatures ranging from 200 to 250 °C. During this stage, all films experienced a significant decrease in weight, primarily due to the thermal decomposition of the glycerol and SA matrix present in the films. However, the incorporation of WEO and ZWP resulted in a smaller decrease in mass compared to the SA films. The DTG results were illustrated in Fig. 5B, where the maximum degradation rate of the SA films corresponds to a temperature of 213 °C, and the temperature of the maximum rate of degradation of the ZWP-S films increases to 215 °C. Overall, the presence of WEO and ZWP did not alter the thermal degradation pattern of the films, however it did result in a minor enhancement in thermal stability. Sahraee, Ghanbarzadeh, Milani, and Hamishehkar (2017) also noted that the inclusion of nanoparticles enhanced the thermal stability of the film.

#### 3.2.6 Moisture content (MC), swelling ratio (SR), water contact angle (WCA) and water vapor permeability (WVP)

The MC, SR, WCA, WVP of the films were given in Table 2. The MC was closely related to the total volume of water molecules occupying the voids in the network structure of the film. The MC of the film was significantly reduced by the addition of WEO and nanoparticles. This is due to the fact that WEO and zein are mainly hydrophobic, with small polarity and low affinity for water, so these have lower MC values, were about 85.95 % and 76.94 % of the MC of pure SA film, respectively. The SR of the films showed a decreasing trend from 2.56 to 1.94. In addition, the barrier property of the films plays a vital role in improving the quality and safety of food products. The WVP of the WEO-S film (3.20 ± 0.08) was significantly lower than that of the SA film (3.64 ± 0.04), which may be related to the hydrophobicity of WEO itself, which is uniformly passed through the polymer chain dispersion, producing hydrophobic sites that can repel water molecules and reduce hydrophilic groups to form hydrophilic bonds, thus improving the barrier properties of the films (Aitboulahsen, El Galiou, Laglaoui, Bakkali, & Hassani Zerrouk, 2020; Cazón, Velazquez, Ramírez, & Vázquez, 2017). The WVP of ZWP-S films was slightly higher than that of WEO-S films. It reported similar findings that the WVP of the production of pectin-based nanocomposite edible films (PGEO@CSNP) combining chitosan nanoparticles and garlic essential oil was higher than that of the garlic essential oil pectin-based films (PGEO) (Dos Santos et al., 2023). This rise could be related to the pore size at the microscopic scale, as well as the intermolecular spacing between the polymer chains at the molecular scale. Further, it is worth mentioning that high values of WCA and hydrophobicity are particularly important for applications focused on food packaging. The WCA of the SA film was 63.24 ± 0.81, which indicates that it was inherently hydrophilic. Upon the addition of WEO and ZWP, the WCA was increased by 8.82 % and 12.70 % respectively, in comparison to the SA film. This significant increment is mainly due to the fact that WEO and zein are hydrophobic substances. Meanwhile, it had been reported that the rough structure of the films may increase the WCA values (Bagheri, Radi, & Amiri, 2019). SEM images confirmed the rough structure of the WEO-S and ZWP-S films (Fig. 1).

#### 3.2.7 Antioxidant properties

**Fig. 6:**
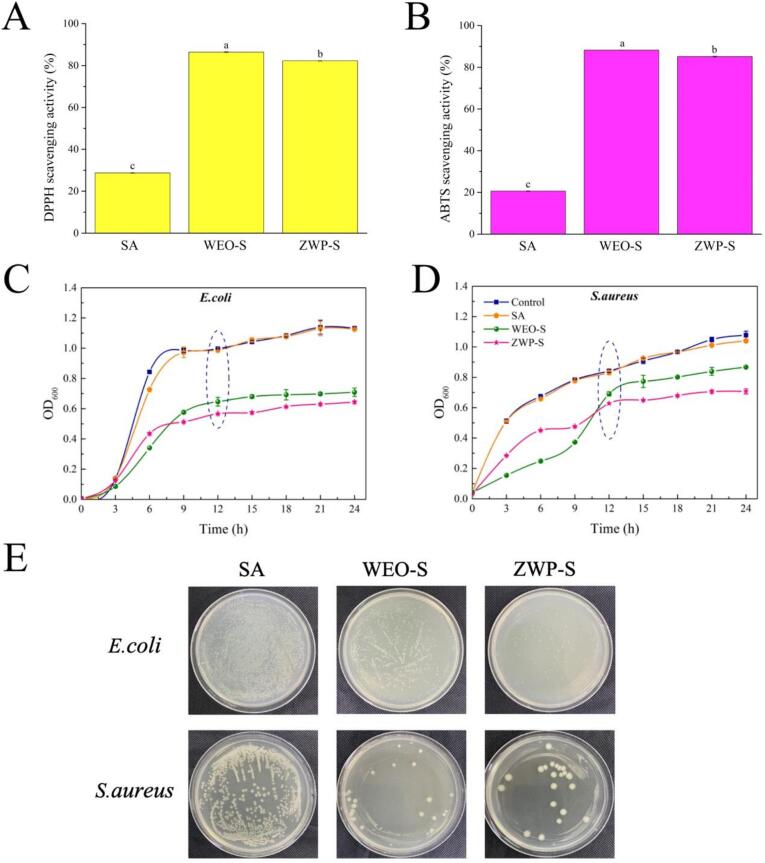
DPPH (A) and ABTS (B) radical scavenging activity of the films. Bacterial growth curves after the films treatments (C, D). Colony growth after 12 h of films treatment (E).

In Fig. 6A, B, the pure SA films exhibited the lowest scavenging capacity for DPPH and ABTS radicals (28.68 % and 20.64 %, respectively), and a similar phenomenon had been reported by Ning et al. (2022). The WEO-S films exhibited superior antioxidant activity due to the presence of components such as monoterpenes and oxygenated monoterpenes in WEO. The superior scavenging ability was particularly evident when comparing the WEO-chitosan-based films with ascorbic acid in a previous study (Liu et al., 2023). The antioxidant activity of the ZWP-S films was slightly reduced, which may be attributed to the effective encapsulation of WEO by the nanoparticles that retarded the release efficiency of the essential oils. In the case of films, the antioxidant activity of EOs is mainly dependent on the interaction with the polymer and the migration of functional active components into the food system, which are dependent on electrostatic interactions, environmental conditions, physical changes, and many other factors (Atarés & Chiralt, 2016).

#### 3.2.8 Antibacterial activity

The SA films had little or no antimicrobial activity against *E. coli* and *S. aureus* in Fig. 6C, D. The addition of WEO and ZWP resulted in a significant decrease in OD_600_ values and growth inhibition of both bacteria throughout all stages. This can be assigned to the inhibitory effects of EOs, which mainly disrupt the lipid structure of cell membranes, including mitochondrial membranes. This disruption leads to the leakage of intracellular material and ultimately causes cell death (Ding et al., 2022). The growth of *E. coli* and *S. aureus* was inhibited by WEO-S films to the maximum extent during the first 6 h. This was due to the high burst effect of WEO in WEO-S during the initial phase. Li et al. (2022) incorporated wampee (*Clausena lansium*) seed EO into chitosan film showed similar enhanced bacterial inhibition against Gram-negative and Gram-positive bacteria. For ZWP-S, under the synergistic protective effect of zein and pectin, WEO was continuously released and the bacteriostatic effect was longer, especially after 12 h. The effect of bacteriostatic activity was more significant. In addition, we found that although WEO have antibacterial properties, their efficacy may decrease after encapsulation and incorporation into the film. The effectiveness effect may also be limited in time. We need to conduct in-depth studies on the durability of the material in future research. Overall. ZWP nanoparticles have the potential to serve as a valuable natural antibacterial agent for the food industry in the future(Kumar, Pratibha, Yadav, Upadhyay, et al., 2023).

#### 3.2.9 Releasability evaluation

**Fig. 7:**
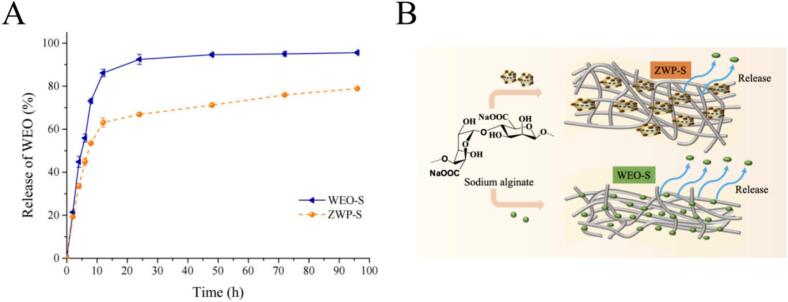
Released curves of WEO from SA-based films (A). Schematic diagram of the possible mechanisms of nanoparticles and incorporation into films for sustained release of WEO (B).

The results were indicated in Fig. 7. The release profiles of the two films could be characterized as two phases of the whole release process, including the initial burst effect and the subsequent sustained release until an equilibrium state was reached during the test period. This was attributed to the gradual migration of WEO from the polysaccharide matrix into the simulated system, and similar results were seen in a previous study (Chen, Lu, Yao, & Pan, 2020). Furthermore, the study revealed that WEO-S demonstrated a substantial burst impact (73 %) with a notably greater release within the initial 8 h. This result was mainly attributed to the collapse of the structure of the polymer membrane, which had a highly hydrophilic structure, and the water-absorbing swelling, and even to some extent solubilization, of the SA base film in the 10 % ethanol solution. The spatial network structure of WEO—S, characterized by weak intermolecular connections, was significantly disturbed in the highly hydrophilic system, resulting in a substantial increase in the release rate of WEO. On the other hand, the release of ZWP-S was slower, possibly because the process of encapsulating WEO with zein and pectin resulted in an 89.65 % encapsulation efficiency, slightly reducing the content of WEO leading to the reduction of the release rate. In addition, as the contact of zein with ethanol disrupts the integrity of ZWP, WEO leaks out of ZWP and migrates into the simulant. It was noteworthy that the intensity of the burst phase of ZWP-S is weakened (53 %) within the first 8 h, out of the nanoparticles' protective mechanism against WEO. In a previous study, Alinaqi, Khezri, and Rezaeinia (2021) determined that polysaccharide films incorporating zein-clove essential oil dissolved well in a 10 % ethanol simulant with prolonged release and a slower process during the first hours of testing. In conclusion, the above results could indicate that ZWP-S has good controlled release properties.

#### 3.2.10 Application of coated film for strawberries freshness preservation

**Fig. 8:**
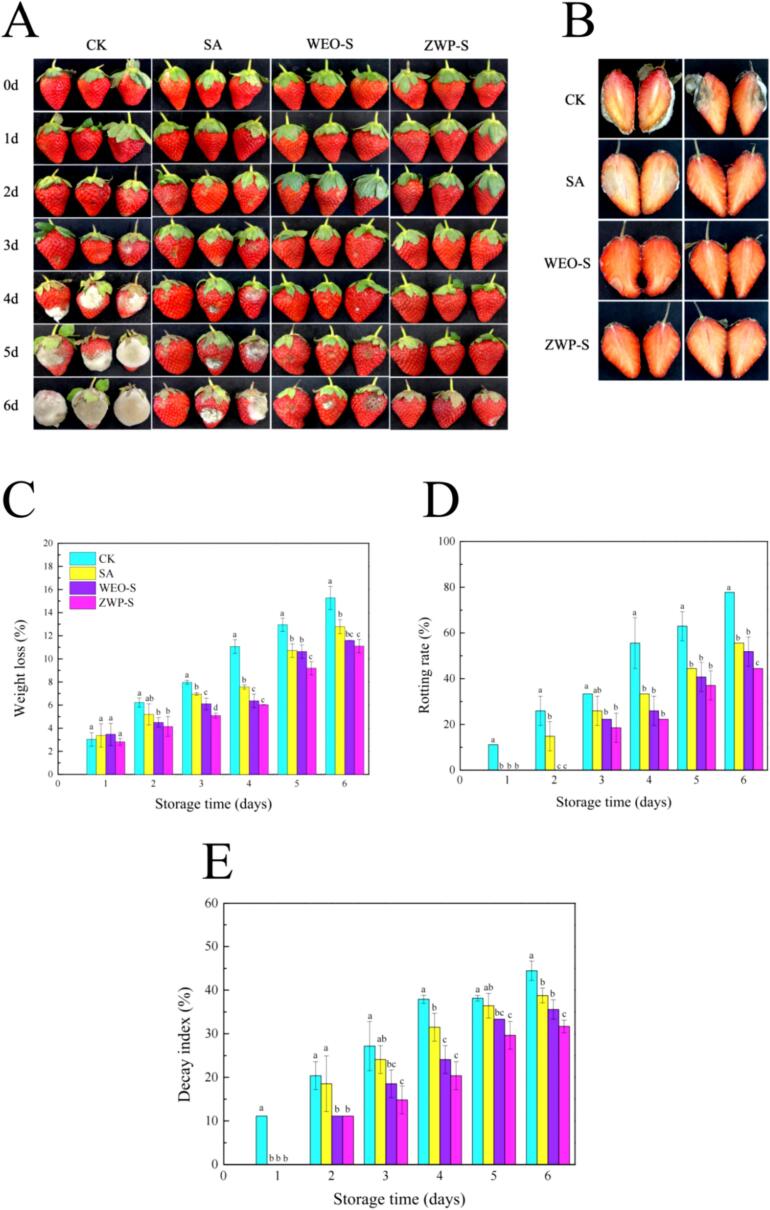
Time-lapse photographs (A), in vivo changes on 6d (B), weight loss (C), rotting rate (D) and decay index (E) of untreated and coated treatments during storage.

The uncoated group showed signs of decay only after 24 h and was usually not accepted for consumption on the 6th day of storage (up to 77.78 % decay rate) in Fig. 8. It is evident that the decay rate of all groups of strawberry samples increased as the storage time extended. The strawberries treated with the freshness-enhancing ZWP-S film solution exhibited the smallest increase in decay rate compared to the uncoated group. This can be attributed to the high hydrophobicity of the bacteriostatic components in the active coatings such as WEO, zein, and the insulating effect of the SA substrate, which protected the strawberries from external factors. In addition, all coated-treated strawberries showed lower weight loss (10.76 %–12.44 %) than the uncoated group (14.26 %) at 6d of storage (Fig. 8C), where WEO released from the nanoparticles slowed down the ripening process of the strawberries more effectively. The zein active packaging treated strawberries with added the EOs were found to have the lowest weight loss (7.35 %) (Ansarifar & Moradinezhad, 2022). At the same time, this was confirmed by the fact that in Fig. 8E, the strawberries treated with ZWP-S film solution presented the lowest decay index (of 31.67). Overall, the ZWP-S film solution coating treatment with slow and controlled release properties leads to longer storage life and marketability of fresh fruits. Similarly, several studies have reported adding edible coatings with plant-active substances and novel nanoformulations to extend the shelf life of vegetables and fruits after harvest (Kumar et al., 2024; Kumar, Pratibha, Trajkovska Petkoska, Gniewosz, & Kieliszek, 2023; Kumar, Rahul, Upadhyay, Gniewosz, & Kieliszek, 2023). Interestingly, we found that the WEO were encapsulated and incorporated into an edible coating that masked the strong flavour of the WEO. When applied to strawberries preservation, the flavour of the strawberries themselves and consumer acceptance were not affected.

## 4 Conclusion

In conclusion, this work prepared ZWP nanoparticles with high encapsulation efficiency (89.7 %). These nanoparticles were then successfully integrated into a film composed of SA. The physical and functional characteristics of the films were analyzed, revealing that the addition of ZWP improved the UV-protective behavior of the pristine films, increased the water penetration pathway, as well as the surface hydrophobicity, improved the mechanical properties, increased the temperature of the maximum rate of degradation, and the density of the structure. Moreover, ZWP-S inhibited the growth of *E. coli* and *S. aureus* while possessing high antioxidant activity (85 %) due to the bacteriostatic component and excellent antioxidant capacity of WEO. Notably, with the synergistic effect of nanoparticles, the physical stability of WEO was enhanced, the volatility was reduced, and ZWP-S possessed a long-lasting release capability. In addition, the coating solution was applied to strawberry preservation, and the results of high-water retention capacity and low senescence assessment could demonstrate that the ZWP-S functionalized coating could maintain strawberry fruit freshness. In summary, this work provides fundamental research on the addition of nanomaterials to edible substrate solutions for the production of efficient active packaging materials and their use in retarding the post-harvest fruit senescence process. Notably, the potential low stability of the material is of crucial importance. SA-based films may exhibit low mechanical or chemical stability, especially in conditions of variable humidity or temperature, which will limit their application in the food industry. In the future, improving material properties will be further explored.

## CRediT authorship contribution statement

**Jinman He:** Writing – original draft, Methodology. **Wanli Zhang:** Writing – review & editing, Methodology. **Gulden Goksen:** Writing – review & editing, Validation, Resources, Methodology, Data curation. **Mohammad Rizwan Khan:** Writing – review & editing, Methodology. **Naushad Ahmad:** Writing – review & editing, Methodology. **Xinli Cong:** Supervision, Resources, Funding acquisition, Conceptualization.

## Declaration of competing interest

The authors declare that they have no known competing financial interests or personal relationships that could have appeared to influence the work reported in this paper.

## Data Availability

Data will be made available on request.

## Appendix A Supplementary data

Table: Graphic Summary Image 1
