# Supplementary material for: Functionalized sodium alginate composite films based on double-encapsulated essential oil of wampee nanoparticles: a green preservation material
Source: Food Chem X. 2024 Sep 19;24:101842. doi: 10.1016/j.fochx.2024.101842 (PMC11456885; doi:10.1016/j.fochx.2024.101842)
Supplement: Supplementary file 1 — Table: Graphic Summary [file mmc1.docx]

Table 1. Characterization of nanoparticles.

| Nanoparticles | Particle size (nm) | Zeta potential (mV) | PDI | Encapsulation Efficiency (%) |
| --- | --- | --- | --- | --- |
| ZWP | 515.9±36.4 | -39.3±0.3 | 0.4±0.1 | 89.7±3.7 |

Table 2. The physical properties of the films.

| Film | SA | WEO-S | ZWP-S |
| --- | --- | --- | --- |
| Thickness（mm） | 0.074±0.004^a^ | 0.066±0.006^b^ | 0.068±0.006^b^ |
| Opacity（A mm^-1^） | 0.87±0.07^b^ | 0.77±0.07^b^ | 2.01±0.42^a^ |
| *L* | 79.96±0.45^a^ | 80.16±0.61^a^ | 77.72±0.21^b^ |
| *a* | 3.24±0.51^a^ | 3.06±0.05^a^ | 3.42±0.13^a^ |
| *b* | -2.11±0.14^b^ | -1.65±0.38^b^ | -0.41±0.65^a^ |
| *△E* | 10.84±0.28^a^ | 10.67±0.51^a^ | 8.38±0.16^b^ |
| Moisture content（%） | 18.43±0.80^a^ | 15.84±0.69^b^ | 14.18±0.73^c^ |
| Water vapor permeability （10^-10^·g·m^-1^·s^-1^·Pa^-1^） | 3.64±0.04^a^ | 3.20±0.08^c^ | 3.35±0.09^b^ |
| Water contact angle（*°*） | 63.24±0.81^a^ | 68.82±3.06^b^ | 71.27±1.67^c^ |
| Swelling ratio | 2.56±0.17^a^ | 2.23±0.10^b^ | 1.94±0.06^c^ |

Values denoted by different letters in each row indicate statistically significant differences at 0.05 confidence level. Data are accompanied by standard errors of the means (n ≥ 3).
